# RETRACTION: Evaluating the Effectiveness of Proactive Perioperative Nursing Strategies on the Prevention of Surgical Site Infections and the Enhancement of Skin Healing in Paediatric Abdominal Surgery

**DOI:** 10.1111/iwj.70667

**Published:** 2025-04-23

## Abstract

**RETRACTION:** YaoX. , XiH. , and DuanW. , “Evaluating the Effectiveness of Proactive Perioperative Nursing Strategies on the Prevention of Surgical Site Infections and the Enhancement of Skin Healing in Paediatric Abdominal Surgery,” International Wound Journal 21, no. 2 (2024): e14735, 10.1111/iwj.14735.PMC1085060942052937

The above article, published online on 07 February 2024, in Wiley Online Library (http://onlinelibrary.wiley.com/), has been retracted by agreement between the journal Editor in Chief, Professor Keith Harding; and John Wiley & Sons Ltd. Following an investigation by the publisher, all parties have concluded that this article was accepted solely on the basis of a compromised peer review process. The editors have therefore decided to retract the article. The authors did not respond to our notice regarding the retraction.

**RETRACTION:** X. Yao , H. Xi , and W. Duan , “Evaluating the Effectiveness of Proactive Perioperative Nursing Strategies on the Prevention of Surgical Site Infections and the Enhancement of Skin Healing in Paediatric Abdominal Surgery,” International Wound Journal 21, no. 2 (2024): e14735, 10.1111/iwj.14735.PMC1085060942052937

The above article, published online on 07 February 2024, in Wiley Online Library (http://onlinelibrary.wiley.com/), has been retracted by agreement between the journal Editor in Chief, Professor Keith Harding; and John Wiley & Sons Ltd. Following an investigation by the publisher, all parties have concluded that this article was accepted solely on the basis of a compromised peer review process. The editors have therefore decided to retract the article. The authors did not respond to our notice regarding the retraction.

